# Dynamic modelling of microRNA regulation during mesenchymal stem cell differentiation

**DOI:** 10.1186/1752-0509-7-124

**Published:** 2013-11-12

**Authors:** Michael Weber, Ana M Sotoca, Peter Kupfer, Reinhard Guthke, Everardus J van Zoelen

**Affiliations:** 1 Leibniz Institute for Natural Product Research and Infection Biology – Hans Knöll Institute, Beutenbergstr. 11a, 07745 Jena, Germany; 2 Department of Cell and Applied Biology, Radboud University, Heijendaalseweg 135, 6525 AJ Nijmegen, The Netherlands

**Keywords:** Gene regulatory network, Network inference, NetGenerator, MicroRNA, Mesenchymal stem cells, Chondrogenesis

## Abstract

**Background:**

Network inference from gene expression data is a typical approach to reconstruct gene regulatory networks. During chondrogenic differentiation of human mesenchymal stem cells (hMSCs), a complex transcriptional network is active and regulates the temporal differentiation progress. As modulators of transcriptional regulation, microRNAs (miRNAs) play a critical role in stem cell differentiation. Integrated network inference aimes at determining interrelations between miRNAs and mRNAs on the basis of expression data as well as miRNA target predictions. We applied the NetGenerator tool in order to infer an integrated gene regulatory network.

**Results:**

Time series experiments were performed to measure mRNA and miRNA abundances of TGF-beta1+BMP2 stimulated hMSCs. Network nodes were identified by analysing temporal expression changes, miRNA target gene predictions, time series correlation and literature knowledge. Network inference was performed using NetGenerator to reconstruct a dynamical regulatory model based on the measured data and prior knowledge. The resulting model is robust against noise and shows an optimal trade-off between fitting precision and inclusion of prior knowledge. It predicts the influence of miRNAs on the expression of chondrogenic marker genes and therefore proposes novel regulatory relations in differentiation control. By analysing the inferred network, we identified a previously unknown regulatory effect of miR-524-5p on the expression of the transcription factor SOX9 and the chondrogenic marker genes COL2A1, ACAN and COL10A1.

**Conclusions:**

Genome-wide exploration of miRNA-mRNA regulatory relationships is a reasonable approach to identify miRNAs which have so far not been associated with the investigated differentiation process. The NetGenerator tool is able to identify valid gene regulatory networks on the basis of miRNA and mRNA time series data.

## Background

Modelling of gene regulatory networks (GRNs) has become a widely used computational approach in systems biology [1]. This development has been greatly promoted by the availability of high-throughput data of adequate amount and quality, such as genome-wide expression data. A major task is the inference of regulatory dependencies between genes on the basis of such data. The inferred gene interactions constitute a network, which contains predictions about cellular regulation. They can motivate the design of new experiments, which might validate predicted dependencies and potentially elucidate unknown regulatory interactions. Successful applications of GRNs have been presented in studies of specific human diseases like cancer [2] and rheumatoid arthritis [3]), murine hepatocytes [4], *E. coli*[5,6] and fungal infection [7].

In this study, we focus on the involvement of microRNAs (miRNAs) in the gene regulation of human mesenchymal stem cells (hMSCs) which differentiate towards chondrocytes. Therefore, we provide a biological background about hMSCs, characteristics and function of miRNAs and modelling approaches which integrate miRNA regulation. HMSCs are multi-potent adult stem cells, which have the capacity to differentiate into multiple cell types, such as chondrocytes, osteoblasts and adipocytes [8,9]. Lineage commitment towards a certain type of cell depends on specific environmental factors. Those factors can activate intracellular signalling pathways which control developmental genes and other signalling pathways. Here, we focus on chondrogenic differentiation, which is characterised by a sequence of intermediate developmental stages, including cell condensation, proliferation, differentiation and hypertrophy [10]. Each of the individual processes is associated with the activity and regulation of lineage-specific genes [11] encoding transcription factors (e.g. SOX9, MEF2C) or ligands of distinct signalling pathways (e.g. TGF-beta1, BMP2, IHH, WNT) [12]. Stimulation of hMSCs by TGF-beta1 initiates the process of chondrogenic differentiation [13]. Although key regulatory genes have been determined, the entire process of regulation in chondrogenesis is still not fully understood. In the recent years, it has become apparent that miRNAs are active regulators in the development of stem cells [14,15].

MiRNAs are short (∼ 22 nucleotides), noncoding RNA molecules, which are able to bind to complementary sequences in target mRNAs, thereby repressing translation or inducing degradation of mRNA molecules [16]. Silencing of gene expression by such post-transcriptional processes has been identified as a new level of gene regulation which is capable of modulating expression levels. In the human genome, more than two thousand mature miRNAs have been identified [17]. Much effort has been put into the revelation of the complex functional network of miRNA and target gene regulation. According to sequence-based predictions, a single miRNA can target hundreds of genes, while a gene can be regulated by multiple miRNAs [18]. Considering the biological function of the target genes, miRNAs were found to regulate various signalling pathways as well as the cell cycle. Interestingly, transcription factor genes are preferred targets of miRNAs [19,20].

Network inference approaches have considered the emerging knowledge about miRNA-dependent regulation by taking account of interactions between miRNAs and mRNAs. Such approaches utilise miRNA target predictions as well as miRNA and mRNA expression data. Consideration of post-transcriptional gene regulation has been contributing to the extension and refinement of GRNs. This new feature has promoted the analysis of dependencies between miRNAs and target genes. For example, tools like MAGIA [21], MMIA [22] and mirConnX [23] perform integrated network analysis on the basis of miRNA target predictions and correlation between miRNA and mRNA expression profiles.

This study aims to present the inference of miRNA regulation as a novel application for the previously published NetGenerator V2.0 tool. The focus of this work is integrated network inference based on mRNA and miRNA time series data and prior knowledge [6,24,25]. The resulting network predicts the role of selected miRNAs in the chondrogenic regulatory network. In comparison to correlation-based approaches (e.g. MAGIA), the NetGenerator tool applies a dynamical model, which is based on linear ordinary differential equations (ODE). Previous network inference studies have successfully shown the ability of linear approaches to result in network models of high biological relevance [4,26-29]. Experimental verification of predicted interactions underscored their validity, while it is often emphasised that biological processes can include more complex relationships [3].

## Results and discussion

### Chondrogenesis data and node selection

**Figure 1:**
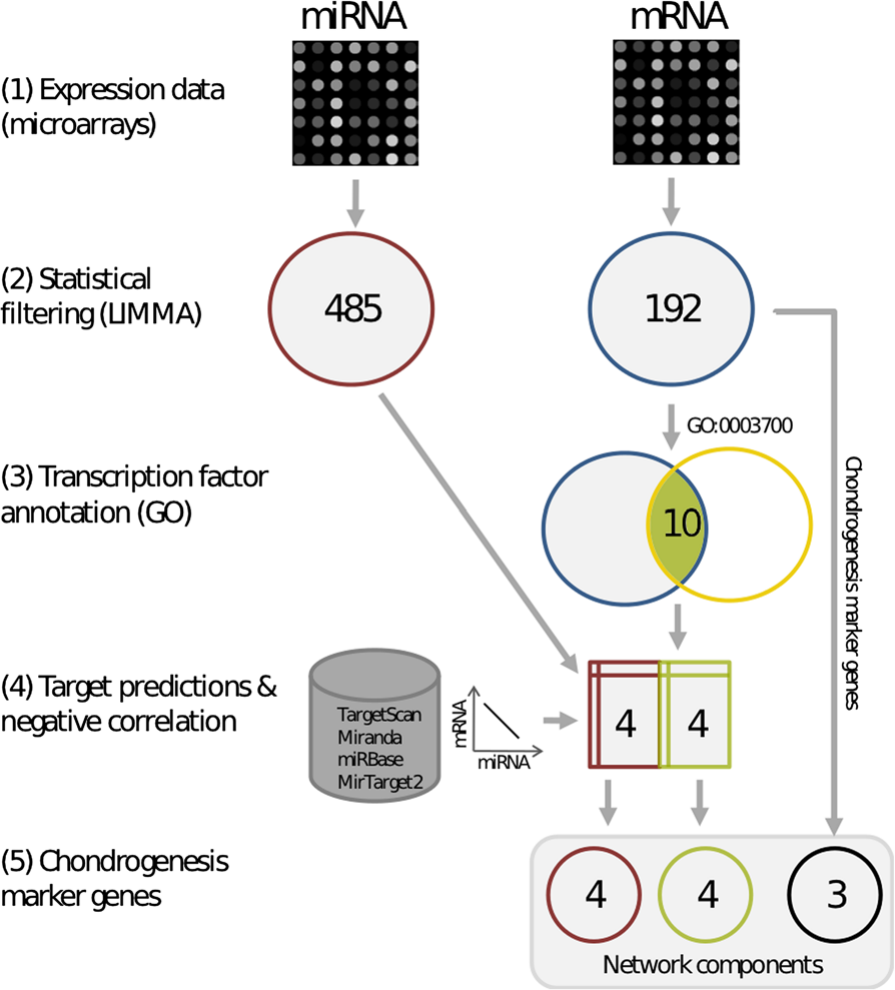
**Gene selection workflow.** This workflow illustrates the steps from pre-processed miRNA/mRNA microarray data to the selection of 11 network components, which includes statistical filtering (2), transcription factor annotation (3), determination of negatively correlated miRNA-mRNA pairs (4) and identification of chondrogenesis marker genes (5).

We analysed a dataset which contained mRNA and miRNA microarray measurements of cultured hMSCs in pellet cultures after stimulation with growth factors TGF-beta1 and BMP2. Both factors are known to induce the process of chondrogenesis [12,13]. Microarray samples were available for 9 time points (0, 3, 6, 12, 24, 48, 72, 120 and 192) h after stimulation, with 3 (mRNA microarray) and 2 (miRNA microarray) replicates per time point. For mRNA microarray data pre-processing, custom chip definition files [30] were used in order to improve the accuracy of the expression estimates. Quantile normalisation was applied to mRNA and miRNA microarray data, respectively (see Methods). This resulted in time series expression data for 12,175 protein-coding genes (mRNAs) and 1,023 miRNAs. Integrated network inference requires the filtering of relevant mRNAs and miRNAs, which constitute the network nodes in the model. A multi-step selection strategy was applied, which included statistical filtering, knowledge-based filtering and time series correlation, to identify miRNAs and genes that are associated with the investigated differentiation process. A workflow which illustrates the sequence of selection procedures, starting from microarray data and resulting in network components, is displayed in Figure 1. Differentially expressed genes (DEGs) and differentially expressed miRNAs (DEMIRs) were identified using the Limma package of the Bioconductor software suite, as described in the Methods section. It resulted in the selection of 192 DEGs and 485 DEMIRs. Subsequently, both sets were used to perform a knowledge-driven selection, which was based on miRNA target predictions and prior knowledge about gene regulation during hMSC differentiation. It is known that the transition from stem cells to terminally differentiated cells is mainly controlled by transcription factors [10]. Moreover, transcription factor genes are reported to be potential targets of miRNAs, because they are significantly overrepresented among the miRNA target genes [20]. Consequently, transcription factor genes from the Gene Ontology term GO:0003700 (sequencespecific DNA binding transcription factor activity) were selected, resulting in 10 differentially expressed transcription factor genes (DETFs). In the next step, predicted interactions between miRNAs and target genes were considered, as they provide a useful subset of linked miRNA and mRNA data. There are numerous resources of experimentally validated or computationally predicted interactions between miRNAs and target mRNAs, such as TarBase [31], miranda [32], miRBase [17], MirTarget2 [33] and TargetScan [18]. Access to these databases is provided by the R package RmiR.Hs.miRNA, which provides the possibility to download the data in form of interaction tables. Taken together, there are more than 1 million predicted interactions stored in these tables. However, most prediction approaches are reported to have relatively low specificity [34]. This issue can be addressed by combining the sequence-based predictions with predictions based on the temporal correlation of miRNA and target genes. To obtain the most reliable interaction predictions, two criteria were applied: (1) at least two of the five above mentioned databases must contain the interaction and (2) the associated expression time series of miRNA and mRNA must be negatively correlated. The first criterion ensures that the considered interactions were found by different approaches. To check the second criterion, the Pearson correlation coefficient between miRNA and mRNA time series was calculated for each interaction pair. Under the assumption that the predicted miRNA target gene interaction is functional, we would intuitively expect a negative correlation coefficient, due to the negative regulatory effect of the miRNA on the expression of its mRNA target. This assumption could be confirmed by Liu et al. [35], who successfully identified miRNA targets by correlation. However, the authors also emphasise that a strong miRNA effect on target gene expression might be better recognisable on target protein level or downstream gene expression levels. Moreover, we noted that positive as well as negative correlations have been reported in the literature for functional miRNA target relations [36]. In this study, we merely focused on strongly negatively correlated miRNA-gene pairs, which can be explained by a repressing interaction between the miRNA and its target. Accordingly, four interactions with a Pearson correlation coefficient smaller than -0.8 were extracted (see Table 1). In other words, focusing on negatively correlated interactions resulted in the selection of 4 DEMIRs and 4 DETFs.

**Table 1:** Predicted miRNA targets and associated time series correlation

**miRNA**	**Gene**	**Method**	**Pearson**
			**correlation**
hsa-miR-494	TRPS1	miranda, mirtarget2, targetscan	-0.89
hsa-miR-298	MEF2C	miranda, mirtarget2	-0.86
hsa-miR-500	SATB2	miranda, mirtarget2	-0.86
hsa-miR-524-5p	SOX9	miranda, mirtarget2	-0.88

Predicted miRNA target genes, corresponding prediction methods and the attained time series correlation.

As reported in the literature, there are prominent chondrogenesis marker genes such as COL2A1, ACAN (aggrecan) and COL10A1, whose expression level indicates the progress of differentiation [37,38]. They encode for structural proteins of the extracellular matrix (ECM) and are differentially expressed in our time series data. Therefore, we added them to the selection of network nodes, because marker genes help to monitor the effects of regulation by miRNAs and transcription factors on chondrogenic differentiation.

In summary, the applied multi-step selection procedure resulted in a set of 11 network components, including 4 miRNAs (miR-524-5p, miR-494, miR-298 and miR-500), 4 transcription factor genes (SOX9, TRPS1, MEF2C and SATB2) and 3 chondrogenic marker genes coding for components of the extracellular matrix (COL2A1, ACAN and COL10A1).

### Network inference

The NetGenerator tool was applied to infer a system of linear ordinary differential equations, which describes a network of regulatory interactions between the components and the influence of the external stimulus (TGF-beta1+BMP2). The general model structure and the utilised optimisation approach is explained in the Methods section. Input data for the tool comprised time series data and prior knowledge about potential regulatory interactions between the components. Time series data were extracted from the available miRNA and mRNA microarray datasets, averaged across replicates at each time point, centered and scaled by their maximum absolute value (see Methods). The resulting time series matrix has 9 rows (time points) and 11 columns (nodes) (Additional file 1). Prior knowledge about regulatory interactions between the nodes was collected from diverse sources, which will be described below.

#### Extraction of prior knowledge

We considered knowledge about the general regulatory potential of each component as well as knowledge about regulatory interactions among the components for GRN inference. For the three component classes ((1) miRNA, (2) transcription factor gene, (3) marker/target gene), prior knowledge regarding the typical biological function was derived as follows: (1) miRNAs primarily function by degradation of their target mRNAs [16]. Therefore, they are expected to downregulate the expression of their respective target genes. (2) Transcription factors positively or negatively regulate the expression of their target genes, which can be protein-coding genes as well as miRNA precursor genes. (3) Genes encoding structural components of the extracellular matrix are not known to have an effect on the expression of neither protein-coding genes nor miRNA genes. Therefore, they were considered to be pure target genes, whose expression is regulated by transcription factors and miRNAs. In addition to this annotation-based knowledge, a set of potential regulatory interactions was obtained from miRNA target predictions, as described in the previous section, and scientific literature. This included four predicted interactions between miRNAs and target genes, which have not been reported in previous studies (see Table 1). To extract regulatory interactions from published work, Pathway Studio V9 was applied, which provides a database of interactions automatically derived from PubMed [39]. In total, four interactions from transcription factors on target genes were retrieved from the database. SOX9 was found to regulate the expression of COL2A1, ACAN and COL10A1 by specifically binding to regulatory elements in the promoter region of these genes [40]. The chondrocyte hypertrophic marker COL10A1 is activated by MEF2C, which binds to conserved sequences in the promoter region of COL10A1 [38]. Finally, the collected prior knowledge was stored in form of interaction matrices (see Methods), which can be processed by NetGenerator.

#### Model inference and interpretation

**Figure 2:**
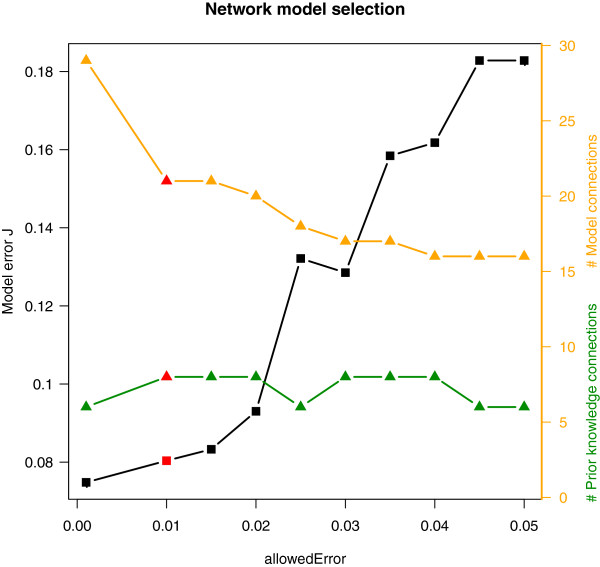
**Network model selection.** A series of ten network inference results, with varying NetGenerator parameter “allowedError”, is shown. For each inference result, the model error J (left ordinate) and the number of model connections / prior knowledge connections (right ordinate, orange/green) are displayed. For further analysis, one model (highlighted in red) was selected (allowedError = 0.01).

**Figure 3:**
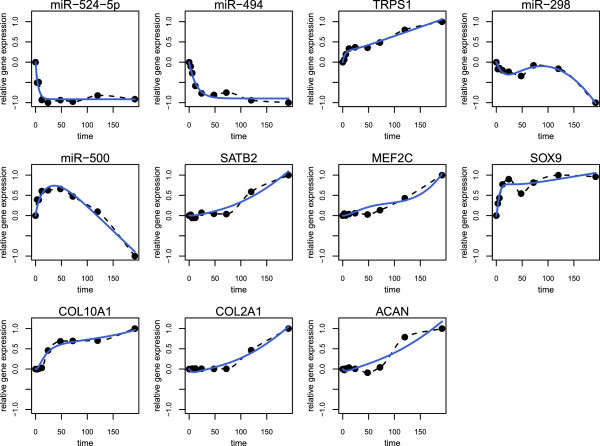
**Chondrogenesis model: time courses.** Comparison of the measured and simulated time courses. Each panel displays the results of one model component: the simulated time course (blue solid line), interpolated measurements (black dashed line) and the measured time series (black dots).

**Figure 4:**
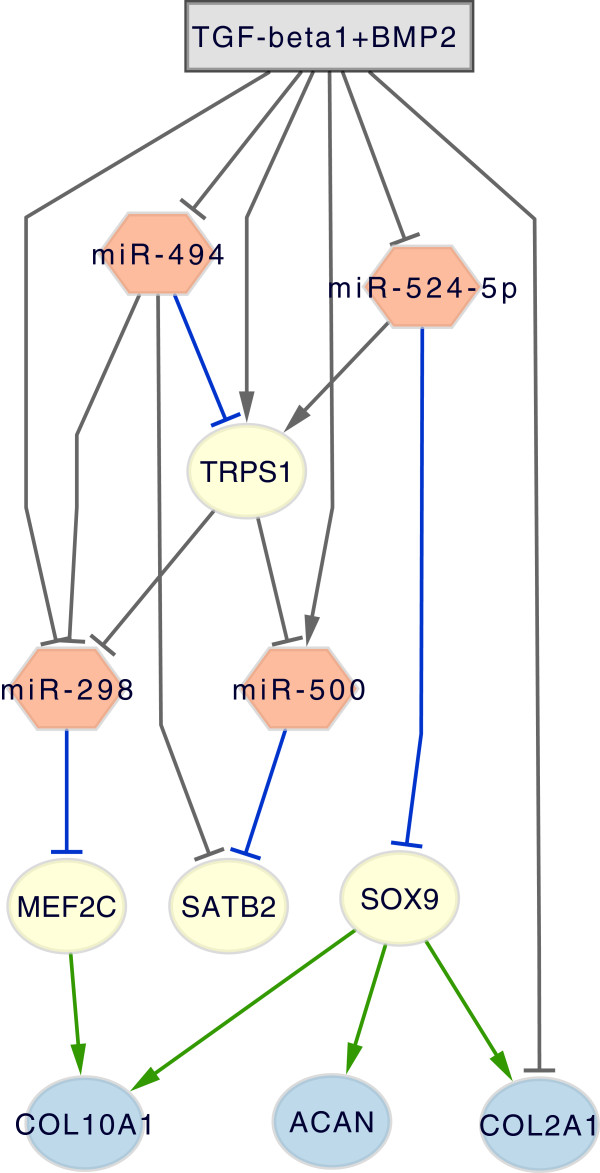
**Chondrogenesis model: inferred network.** Network structure of the chondrogenesis model, which contains the input TGF-beta1+BMP2 and 11 nodes. Nodes represent either a miRNA (miR-524-5p, miR-494, miR-298, miR-500), a transcription factor gene (SOX9, MEF2C, TRPS1, SATB2) or a chondrogenic marker gene (COL2A1, COL10A1, ACAN). Connections are coloured in green (consistent with prior knowledge), blue (predicted miRNA target site) and black (predicted interaction).

The NetGenerator approach aims to identify a set of model parameters which is optimal with respect to the given input data and the presumption of a sparse interaction matrix. The algorithm’s objective is to minimise the model error J, which quantifies the deviation between measured and simulated data, and to consider prior knowledge. The balance between network complexity and an adequate model error is controlled by the parameter “allowedError”, which is the permitted maximum error for each time series. A series of inference results varying this parameter was analysed with respect to the model error, the model complexity (total number of connections) and the number of correctly integrated known connections (see Methods and Figure 2). This resulted in the selection of an optimised model, which shows a good fit to the measured time series data (J=0.0833) and contains 8 known interactions. Simulated and measured time series are compared in Figure 3. The simulated time courses (blue lines) show a good reproduction of the measured time series data (black points). Even if the model seems to be excellent in terms of the objectives mentioned above, a model validation is necessary. The main reason is to prevent over-fitting of the measured time series data by the model. Therefore, the model’s robustness against noise in the data was evaluated by using an approach which is based on repeated resampling of the time series data (see Methods). This procedure resulted in a table of occurrence frequencies for each interaction of the optimised model (Additional file 2). While most of the connections attained a high frequency, two connections with a frequency less than 40% were discarded from the network. All remaining interactions were considered robust against minor fluctuations in the expression data. The final network (Figure 4) consists of 11 nodes, thereof 4 miRNAs, 4 transcription factors (TF) and 3 target genes (chondrogenesis marker), and of the external stimulus (TGF-beta1+BMP2). There are 19 stable connections in the network, which indicate transcriptional or post-transcriptional regulation, depending on the type of the connected components. Considering the proportion of nodes and connections (11 nodes / 19 connections) the network appears to be sparsely connected. There are 6 input-to-node and 13 node-to-node interactions. The latter type of interactions can be further grouped into miRNA-miRNA (1), miRNA-TF (6), TF-miRNA (2) and TF-target (4) interactions. Four connections are coloured in green, which indicates their concordance with literature knowledge. Four connections are coloured in blue, because they are underpinned by predicted miRNA target sites. Connections coloured in black represent hypothetical regulatory interactions without further evidence.

#### Biological interpretation

The biological interpretation of the network will be based on transcription factor nodes (SOX9, MEF2C, TRPS1, SATB2), by identifying regulator and target nodes for each of them. This promotes the understanding of the model, particularly of the mechanism that enables miRNAs to interfere with transcriptional regulation in order to control the differentiation process. As seen in the final model, the input stimulus (TGF-beta1+BMP2) inhibits the expression of 3 miRNAs (miR-494, miR-524-5p, miR-298) and activates miR-500, which is in turn suppressed by TRPS1. Consequently, the negative effect of downregulated miRNAs on their target genes is attenuated, which leads to the activation of the transcription factor genes SOX9, MEF2C, TRPS1 and SATB2. SOX9, the main regulatory factor in chondrogenesis [41], is inhibited by miR-524-5p, a finding which is supported by a predicted miRNA target site (Table 1). Since miR-524-5p expression is suppressed by the TGF-beta1+BMP2 stimulus, SOX9 expression increases and leads to activation of the differentiation markers COL2A1, ACAN and COL10A1. This transactivation is enabled by the presence of a consensus binding motif ((A/T)(A/T)CAA(A/T)G), which is shared by the SOX family members [40]. In COL2A1, multiple copies of this motif could be identified in an enhancer located in intron 1. Activation of ACAN could be associated with the binding of SOX9 in its first intron [42] and the COL10A1 promoter contains a distal enhancer element 4.3 kb upstream of the transcription start site [41]. Therefore, primary chondrogenesis might be under control of miR-524-5p through the modulation of the expression of SOX9 and its target genes. The MADS box transcription factor MEF2C, which controls chondrogenic hypertrophy, positively regulates expression of COL10A1 through binding to conserved sequences in the promoter region [38]. Negative regulation of MEF2C by miR-298 might be a mechanism to prevent early activation of hypertrophic genes. The transcriptional repressor TRPS1 is known to be activated by a specific type of BMP-signalling and promotes chondrogenic differentiation by transcriptional repression of only a few known target genes [43]. In our model, its expression is regulated by the stimulus as well as by miR-494 and miR-524-5p. The interaction with miR-494 is underpinned by prior knowledge (blue connection in Figure 4), but surprisingly there is also a predicted binding site for miR-524-5p within the TRPS1 mRNA. However, the positive sign of the connection suggests that the assumed inhibitory effect may be not reflected by the given data. In recent literature, extensive control of TRPS1 by at least 7 miRNAs has been described for the process of skeletal development [44]. In the network, TRPS1 inhibits the expression of miR-500 and miR-298, which controls the chondrogenic transcription factor MEF2C. While knowledge about target genes of TRPS1 is rare, it is known that TRPS1 can upregulate the chondrogenic marker gene COL10A1 and thereby promote chondrogenic differentiation [43]. SATB2, a transcription factor mainly associated with osteogenesis [11], is repressed by miR-500 and miR-494, as predicted by the model. A potential regulation of SATB2 by miR-500 is supported by the associated binding sequence (Table 1). However, since there is no influence of SATB2 on the expression of chondrogenic marker genes in the network, it is less relevant for chondrogenesis according to our model.

To summarise, the involvement of transcription factor genes is a central part of the model. The model integrates transcriptional regulation (by transcription factor genes) and post-transcriptional regulation (by miRNAs) and thereby displays the interrelationship between miRNAs and transcription factors. Since all four investigated miRNAs are ultimately downregulated, the model proposes the suppression of miRNA activity, which gives rise to the activation of the transcriptional regulators of chondrogenic differentiation such as SOX9. The model comprises miRNAs acting on different stages of the differentiation process including early proliferation and late hypertrophic stages. The downregulation of miR-524-5p provides an interesting explanation about how chondrogenic differentiation might be modulated on the level of post-transcriptional mRNA interference. Furthermore, we found expression of miR-524-5p to be differently regulated during osteogenic and adipogenic hMSC differentiation (Additional file 3). This indicates that the repression of miR-524-5p activity may be relevant for lineage specificity during hMSC differentiation. Previous studies have reported that miR-524-5p is active in glioma cells and interacts with two components of the Notch signalling pathway [45].

### Experimental validation

**Figure 5:**
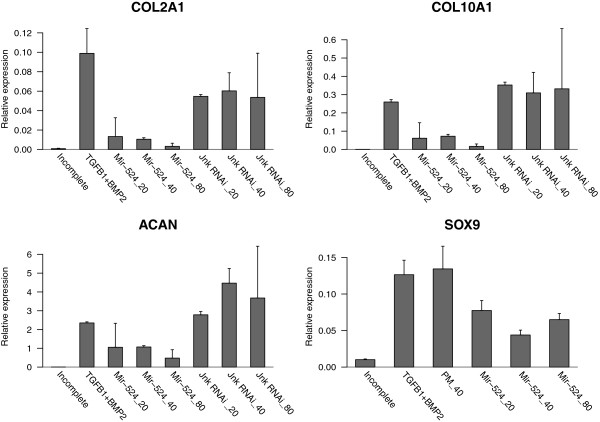
**Experimental validation.** Barplots depict the relative expression of COL2A1, COL10A1, ACAN and SOX9, respectively, under a series of distinct conditions. Those include untreated cells (Incomplete), TGF-beta1+BMP2-treated cells (TGFB1+BMP2), lentiviral based miR-524-5p overexpression with three different concentrations (Mir-524_20, Mir-524_40, Mir-524_80) and negative control experiments (Jnk RNAi_20, Jnk RNAi_40, Jnk RNAi_80, PM_40).

The inferred chondrogenic network implies that mir-524-5p is able to target SOX9 mRNA, thereby inhibiting expression of SOX9 and its target genes (COL2A1, ACAN, COL10A1). Therefore, we performed overexpression experiments of mir-524-5p in hMSCs to validate if chondrogenesis is impaired in this case. Changes in chondrogenic differentiation were measured based on the expression of specific marker genes. For this, hMSCs were transfected with lentivirus harbouring the mir-524-5p coding sequence, while a non-related murine Jnk RNAi lentivirus was used as a negative control. Then, hMSCs were allowed to differentiate for 14 days into chondrocytes after which the expression of chondrogenic marker genes was measured by using qPCR. Relative expression of marker genes of transfected cells was compared to the negative control (incomplete: differentiation in culture medium without any growth factors added) and a positive control (TGFB1+BMP2: medium containing TGF-beta1 and BMP2) in which differentiation occurs in culture medium but without lentiviral transduction (see Figure 5). The positive control sets the baseline for comparison of the different expression levels, because differentiation is optimal. The results showed that mir-524-5p overexpression decreases the relative expression of all measured marker genes. The decrease in relative expression is significantly stronger than the decrease observed when using the non-related murine Jnk RNAi lentivirus. This negative control had no effect on chondrogenesis observed for all marker genes tested. In addition, hMSCs were transfected with mir-524-5p lentivirus and the empty pMIRNA backbone (PM_40) vector lentivirus (as negative control) and, subsequently, cells were allowed to differentiate for 14 days prior RNA qPCR analysis. The relative expression of SOX9 decreased when mir-524-5p was overexpressed and the control virus (PM_40) remained comparable to the relative expression of the positive control (TGFB1+BMP2). In conclusion, experimental validation showed that lentiviral based overexpression experiments of mir-524-5p in differentiating hMSCs resulted in a significant inhibition of several chondrogenic marker genes compared to either non-transfected hMSCs or transfected with a control lentivirus.

## Conclusions

This study presented and analysed large-scale miRNA time series expression data, which captures the post-transcriptional level of chondrogenic differentiation of hMSCs. The combination of miRNA and mRNA microarray data enabled the identification of miRNAs which potentially act in this developmental process. To detect the most relevant miRNAs, a custom filtering based on diverse biological prior knowledge (literature knowledge, transcription factor annotation, miRNA target predictions) in conjunction with statistical criteria was applied. Four miRNAs (miR-524-5p, miR-494, miR-298 and miR-500) were found to be potentially involved in the regulation of chondrogenesis. To infer an integrated network of miRNA and gene regulation, we presented a novel application of the NetGenerator tool i.e. its capability to integrate miRNA and mRNA time series data into a single network inference. The good quality of the resulting network model with regard to complexity, data fit and robustness underlines the tool’s utility to infer the post-transcriptional level of gene regulation. Analysis of the network resulted in hypotheses and additional experiments which verified model predictions by showing that miR-524-5p can affect the expression of the central transcription factor gene SOX9 and differentiation marker genes. Therefore, this work demonstrated how dynamic modelling of miRNA regulation can enhance the understanding of a specific biological process and lead to the discovery of new regulatory interactions.

## Methods

### Culture and differentiation of human mesenchymal stem cells

Human mesenchymal stem cells (hMSCs), harvested from normal human bone marrow, were purchased from Lonza (Walkersville, MD) at passage 2. Cells were tested by the manufacturer and were found to be positive by flow cytometry for expression of CD105, CD166, CD29 and CD44 and negative for CD14, CD34 and CD45. We confirmed multipotency of all donor batches based on in vitro osteo-, chondro- and adipogenic differentiation capacity [9]. The cells were expanded for no more than 5 passages in ‘mesenchymal stem cell growth medium’ (MSCGM, Lonza, Walkersville, MD) at 37°C in a humidified atmosphere containing 7.5% CO_2_. Studies were performed with hMSCs from multiple donors, including 5F0138, 5F0138 and 1F1061. For chondrogenic differentiation, hMSCs were trypsinised and 2.5x 10^5^ cells pelleted in a 10 ml round bottom tube (Greiner Bio-One, Monroe, NC) for 10 min at 250xg. Cell pellets were subsequently cultured for 21 days in chondrogenic differentiation medium, consisting of proliferation medium supplemented with 6.25 µg/ml insulin, 6.25 µg/ml transferrin, 6.25 ng/ml sodium selenite, 5.35 µg/ml linoleic acid, 400 µg/ml proline, 1 mg/ml sodium pyruvate, 10^-7^ M dexamethasone, 50 µg/ml sodium L-ascorbate (all obtained from Sigma-Aldrich, St. Louis, MO), in the absence (incomplete or control) or presence of 10 ng/ml recombinant TGF-beta1 in combination with 50 ng/ml recombinant human BMP2 (TGF-beta1+BMP2). Growth factors were obtained from R&D Systems.

### mRNA and microRNA profiling

Affymetrix Human Genome U133A (HG-U133A) microarrays were employed in triplicate experiments at 9 time points (0, 3, 6, 12, 24, 48, 72, 120 and 192 hours after onset of treatment with TGF-beta1+BMP2). Further experimental details can be found in [9]. For miRNA profiling, 18 RNA samples were obtained from duplicate experiments, one biological condition and measured at 9 time points (0, 3, 6, 12, 24, 48, 72, 120 and 192 hours after onset of treatment with TGF-beta1+BMP2). RNA was extracted using TRIzol ^*Ⓡ*^ according to the protocol provided by the manufacturer (Invitrogen). For each sample, 5 µg of RNA was used for miRNA profiling. Hybridisation and profiling were performed using Exiqon (Vedbaek, Denmark) capture probe sets spotted on Schott Nexterion Hi-Sense E glass slides [46].

### Determination of relative expression levels of chondrogenic marker genes using quantitative PCR (qPCR)

Total RNA was isolated from chondrogenic pellets using the Mirvana (Ambion) kit according to manufacturer’s instructions. The isolated total RNA (≈100 ng) was then used as a template in a 20 µl reverse transcriptase reaction using superscript reverse transcriptase from Invitrogen according to manufacturer’s instructions using random hexamers to prime the reaction. The following cycling conditions were used: 10 min at 20°C, 45 min at 42°C and 10 min at 94°C. The resulting cDNA solution was diluted 5x by adding 80µl water. qPCR of chondrogenic markers was performed using the following human primers: COL2A1 (forward: 5’-CTGCCAGTGGGCAACCA-3’, reverse: 5’-TTTGGGTCCTACAATATCCTTGATG-3’), COL10A1 (forward: 5’-AAAGCTGCCAAGGCACCAT3’ and reverse: 5’-AGGATACTAGCAGCAAAAAGGGTATT-3’), ACAN (forward: 5’-GACAGAGGGACACGTCATATGC-3’ and reverse: 5’-CGGGAAGTGGCGGTAACA-3’) and SOX9 (forward: 5’-GCAAGCTCTGGAGACTTCTGAAC-3’ and reverse: 5’-ACTTGTAATCCGGGTGGTCCTT-3’), expression values were normalised and corrected using RPS27a housekeeping gene (Forward: 5’-GTTAAGCTGGCTGTCCTGAAA-3’ and reverse: 5’-CATCAGAAGGGCACTCTCG-3’). Relative expression was calculated using the following formula: Relative expression: 2^-*Ct*^·10^6^ marker gene / 2^-*Ct*^·10^6^ RPS27a. Data are presented as a fraction of RPS27a expression and all qPCRs were performed in duplicates (Additional file 4).

### Microarray data analysis

Microarray data pre-processing and network inference were entirely performed in the statistical programming environment R [47] using Bioconductor software tools [48]. Pre-processing aims to remove non-biological noise from the data and to estimate gene expression levels.

#### Pre-processing of mRNA microarray data

Data from mRNA microarray experiments were pre-processed using the customised chip definition package “gahgu133a” and the robust multi-array average (RMA) procedures [49]. The chip definition package provides custom probe-sets for the Affymetrix HG-U133A chip, which reduces the number of cross-hybridising probes [30]. The remaining probes allow for a one-to-one correspondence between probe-set and gene. RMA procedures were applied for background correction, quantile normalisation and summarisation. The resulting signal matrix contains the logarithmised gene expression estimates for 12,175 genes.

#### Pre-processing of miRNA microarray data

First, mean signal values were extracted for each of the measured miRNAs. Secondly, quantile normalisation was applied, which is provided by the RMA package. This led to logarithmised miRNAs expression estimates for 1,023 miRNAs. In contrast to mRNA microarray data, there can be multiple probe-sets representing the same miRNA.

### Statistical filtering

We applied the LIMMA package of the Bioconductor software suite [50] to the miRNA and the mRNA dataset, respectively. It provides routines using an empirical bayes approach for the identification of differentially expressed genes. Time series data can be analysed by contrast terms, which were defined by subtracting the control group from the stimulus group at each time point. Statistical significance was determined by applying a moderated F-statistics. Finally, LIMMA returned a ranked table, which contains columns for gene name, fold-change and adjusted p-values. While for mRNA selection a 2-fold-change criterion was combined with a p-value threshold (Benjamini-Hochberg adjusted p-value ≤10^-10^), miRNA selection was merely based on a 2-fold-change criterion, due to the low replicate number in the miRNA dataset (2 replicates per time point).

### Time series standardisation

Time series standardisation is a pre-processing step required by the NetGenerator tool [25]. It includes centering and scaling of each time series. Centering implies subtraction of the first value from all values such that the transformed time series starts from zero. Subsequent scaling divides the centered time series by its maximum absolute value, which leads to gene-wise scaled data varying within -1 and 1.

### Network inference

Network inference was performed using the NetGenerator tool, which models gene regulation by a system of ordinary differential equations (Equation 1).

(1) $${\dot{x}}_{i}(t)=\overset{N}{\underset{j}{\sum }}a_{i,j}x_{j}(t)+b_{i}u(t)$$

Dynamic change of expression *x*_*i*_ of component *i* is described by the sum of weighted gene expressions of *N* genes and the weighted input *u*(*t*), which is a stepwise constant function representing the external stimulus (e.g. TGF-beta1+BMP2). The values of *x*_*i*_ can be interpreted as standardised expression changes of component i between stimulated and non-stimulated (control) state, which serves as a reference point.

Regulatory interactions are modelled by the interaction parameters *a*_*i*,*j*_ and the input parameters *b*_*i*_. A positive parameter value denotes an activating connection, a negative value denotes an inhibitory connection and the value zero denotes no connection. Consequently, the GRN structure is determined by the model’s interaction parameters, which have to be identified by the NetGenerator algorithm. The algorithm’s central part is a heuristic algorithm, which performs network structure and parameter optimisation. Structure optimisation applies the principle of sparseness. Iterative development of sparse sub-models explicitly restricts the number of identified connections. In each development step, parameter optimisation is applied to obtain interaction and input parameter values. The resulting model contains a minimal number of parameters that is necessary to obtain a good fit between simulated model and measured time series. A more detailed description of the algorithm can be found in [6,24,25].

NetGenerator also allows for integration of additional information about regulation between the components, referred to as prior knowledge. As this knowledge is independent of the time series data, it represents valuable additional input for the network inference. NetGenerator is capable of using prior knowledge during the structure optimisation process, while also dealing with contradictions between prior knowledge and time series data. Knowledge is provided in form of an interaction matrix which contains values assigned to particular connections, coded in the following way: no connection (0), activation (10), inhibition (-10), activation or inhibition (1) or not available (NA). NetGenerator provides a flexible integration mode which ignores prior knowledge in case the model fit is worsened.

Since NetGenerator contains a heuristic core, it depends on the setting of configuration parameters. The central parameter “allowedError” controls the permitted total deviation between simulated and measured data for each time series. To achieve an optimal result, we performed a series of network inference runs varying the value of this parameter (0.001, 0.01 (0.005) 0.05) resulting in ten models (see Figure 2). The resulting models were assessed on the basis of the actual model error J and the number of successfully integrated known connections. An optimal model reproduces the data with a low error (high accuracy), while attaining a relatively low model complexity (number of interactions). Considering the ten models, we found the second model (allowedError=0.01) to be optimal with respect to model error (J=0.0833), model complexity (21 interactions) and integrated prior knowledge connections (8). The network model is shown in Figure 4 and simulated time courses are shown in Figure 3.

For model validation, robustness of the inferred network against small distortions of the time series data was tested. Inaccuracy may occur in the data due to technical or biological variance. A robust inference result is expected to maintain a similar network structure when the input data is slightly perturbed. Therefore, we applied random perturbation of the time series data by sampling from a Gaussian noise distribution ($N(0,\;0.05^{2})$) and subsequent network inference. This procedure was repeated 100 times leading to a series of models, from which relative frequencies for each of the connections of the final model were derived. Connections which were inferred with a frequency of at least 50% were considered stable and therefore reliable.

### Maintenance, lentiviral transfection and induced chondrogenesis of hMSCs

hMSCs were maintained in DMEM medium supplemented with 10% FBS, 1% pyruvate, 1% L-glutamine, 100 U/ml penicillin and 100 µg/ml streptomycin (referred to as proliferation medium, PM) and incubated at 37°C and an humidified atmosphere containing 7,5% CO_2_. The day before lentiviral transduction, about 5·10^5^ cells were transferred to 25 cm^2^ flasks in PM and incubated for 18 hours, as before. Then, cells were transfected using lentivirus containing either the empty pMIRNA backbone vector (control) or pMIRNA vector with mir-524-5p premature DNA sequences (purchased from System Biosciences). Lentiviruses were added in various concentrations (20 ng, 40 ng and 80 ng virus/30.000 cells) in addition to 1 mg/L polybrene (Milipore). The transfected cells were incubated for 2-3 days to allow for lentiviral integration and expression of the introduced transgenes. Transfected hMSCs were grown as pellets (by centrifugation) in high-glucose DMEM supplemented with 100 U/ml penicillin, 100 µg/ml streptomycin, 1% L-glutamate 6,25 µg/ml insulin, 6,25 ng/ml sodium selenite, 6,25 µg/ml transferrin, 5,35 µg/ml linoleic acid, 400 µg/ml proline, 1% pyruvate, 100 nM dexamethasone, 50 µg/ml sodium ascorbate and 1,25 mg/ml bovine albumin (listed compound from Sigma). This medium will be further referred to as incomplete medium. Differentiation experiments were performed using incomplete medium in the presence or absence of 10 ng/ml TGF-beta1 and 50 ng/ml BMP2 (both purchased from R&D Systems). Differentiation of hMSCs chondrogenic pellets was allowed for 14 days.

## Competing interests

The authors declare that they have no competing interests.

## Authors’ contributions

MW and AS drafted the manuscript. MW performed pre-processing and modelling of the network. PK contributed to the network component selection. AS and EJvZ contributed to experimental set-ups, measurements and biological interpretation of the network. All authors read and approved the final manuscript.

## Supplementary Material

Additional file 1 **Expression data of network components.** Expression data, which contains non-standardised expression differences between stimulation and control of the corresponding 11 network components. Click here for file

Additional file 2 **Table of connection frequencies from model validation.** A table of the network model connections and their relative frequencies in the model validation. Click here for file

Additional file 3 **Time series of miR-524-5p expression.** Time series of miR-524-5p expression after chondrogenic, osteogenic and adipogenic stimulation of human mesenchymal stem cells. Click here for file

Additional file 4 **Expression data for network validation.** Expression data of the validation experiments which investigated the impact of miR-524-5p overexpression on the expression level of SOX9, COL2A1, COL10A1 and ACAN. Click here for file
