# Supplementary figures and images for: Dynamic modelling of microRNA regulation during mesenchymal stem cell differentiation
Source: BMC Syst Biol. 2013 Nov 12;7:124. doi: 10.1186/1752-0509-7-124 (PMC4225824; doi:10.1186/1752-0509-7-124)

## Time series expression of miR-524-5p

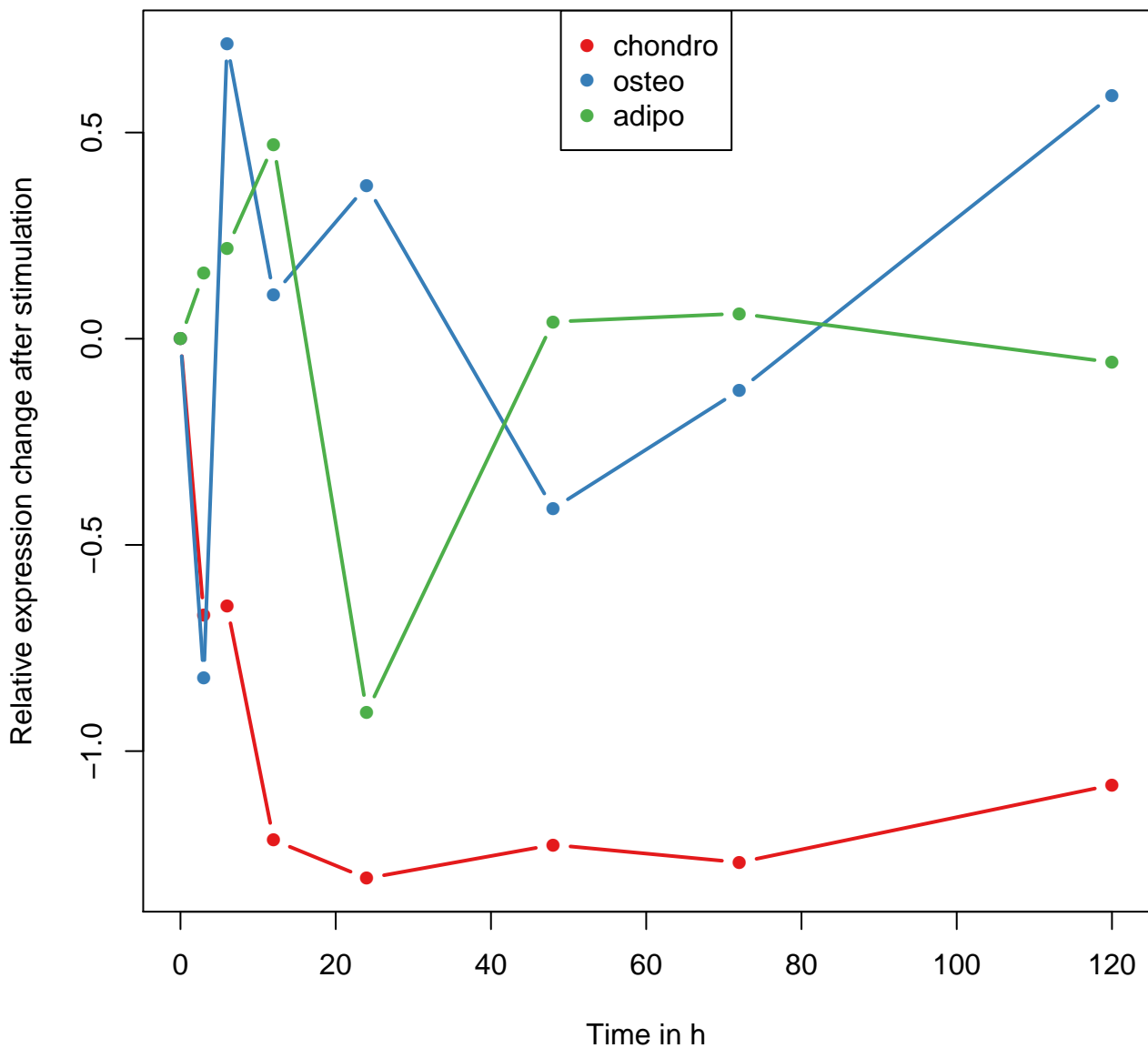

Supplement: Additional file 3 — Time series of miR-524-5p expression. Time series of miR-524-5p expression after chondrogenic, osteogenic and adipogenic stimulation of human mesenchymal stem cells. [file 1752-0509-7-124-S3.pdf]
